# COST Action FP0905: Biosafety of forest transgenic trees: improving the scientific basis for safe tree development and implementation of EU policy directives

**DOI:** 10.1186/1753-6561-5-S7-P185

**Published:** 2011-09-13

**Authors:** Crstina Vettori, Matthias Fladung

**Affiliations:** 1 Plant Genetics Institute UOS FI – CNR, Via Madonna del Piano 10, 50019 Sesto Fiorentino FI, Italy; 2 Johann Heinrich von Thuenen Institute (vTI), Institute for Forest Genetics, Sieker Landstr. 2, D-22927 Grosshansdorf, Germany

## Background

The potential for unintended consequences of the spread of foreign genes (either via vertical or horizontal transfer) and of pleiotropic effects following transgene expression may be enhanced in long-lived forest trees. The COST Action FP0905 will focus on four key aspects related to the biosafety of genetically modified trees (GMTs): (a) analyses of the efficiency of existing gene containment strategies to avoid or if not possible to minimize gene flow; (b) facilitate efforts to develop site-specific integration of transgenes in tree genomes to minimize variability of transgene expression and pleiotropic effects, (c) evaluate possible methods to monitor GMTs in the whole production chain, and (d) conduct socio-economic and cost/benefit analyses in relation to the use of GMTs in plantations.

This Action combines multidisciplinary knowledge generated with transgenic lines of different forest genera (such as, *Populus* spp., *Pinus* spp., *Eucalyptus* spp., *Betula* spp., *Castanea* spp., *Picea* spp., etc.) as well as extensive expertise in correlated topics. The information gained should contribute to strengthen the scientific basis for the execution of the EU policy directives related to transgenic trees intended for cultivation in Europe. The main objective of the COST Action is to evaluate and substantiate the scientific knowledge relevant for GMT biosafety protocols by putting together already existing information generated in various European and Non-EU countries as basis for future EU policy and regulation for the environmental impact assessment and the safe development and practical use of GMTs.

## Work plan and organisation

To reach its aim, the work plan of the Action is organised in 4 Working Groups (WGs) to implement collaboration of scientists.

WG 1 - Biological characterization of GMTs: to characterize the GMTs in respect to their genetic and phenotypic features relevant for gene flow, gene containment and gene targeting.

WG 2 - Environmental impact assessment and monitoring of GMTs in the whole production chain from plantation to final products: to study environmental risk assessment strategies and monitoring the GMTs along the whole production chain.

WG 3 - Socio-economic implications of and recommendations for the use of GMTs: to make socio-economics analyses of the use of GMTs considering the concerns and acceptance by the public, the economic potential for GMTs and R&D efforts to be invested, as well as cost/benefit analyses, and propose recommendations for the use of GMTs.

WG 4 - Management of intranet - internet websites and dissemination: through a website (www.cost-action-fp0905.eu), provide science-based information and increase public awareness in the utilization of GMTs in forest plantation and at the same time safeguarding the environment

The knowledge gained will be summarised in a book as a final output of this Action which will report the state of art of knowledge and research on GMTs with suggestion on how to effectively implement present EU directives on GMO considering the problematic of forest trees and their environmental impacts.

The Action started the 12th of April 2010 and it will end the 11th of April 2014. Actually, 23 COST countries (Austria, Belgium, Bosnia and Herzegovina, Bulgaria, Croatia, Denmark, Estonia, Finland, France, Germany, Greece, Israel, Italy, Latvia, Netherlands, Norway, Romania, Serbia, Slovak Republic, Slovenia, Spain, Sweden, and United Kingdom) have signed the Memorandum of Understanding (MoU). Seven NON-COST countries (Albania, Australia, Canada, China, New Zealand, South Africa, USA) are participating to the Action.

## Conclusion

With integration of all the countries listed above, the EU COST Action FP0905 is expected to generate important benefits as it also foresees a strong collaboration among R&D bodies and legislative directives. This kind of collaboration will be fundamental, on the one hand, to address policy-making efforts and, on the other hand, to allow the scientific community to discuss to public concerns in a responsible way, particularly concerning socio-economic implications and biosafety issues of transgenic tree plantations.

